# Rhinosinusitis and Aspirin-Exacerbated Respiratory Disease

**DOI:** 10.1155/2012/273752

**Published:** 2012-07-04

**Authors:** Maria L. Garcia Cruz, M. Alejandro Jimenez-Chobillon, Luis M. Teran

**Affiliations:** ^1^Department of Immunogenetics and Allergy, Instituto Nacional de Enfermedades Respiratorias, 14080 DF, Mexico; ^2^Otolaryngology Department, Instituto Nacional de Enfermedades Respiratorias, 14080 DF, Mexico

## Abstract

Rhinosinusitis is a feature of aspirin-exacerbated respiratory disease (AERD), which in the initial phase is manifested as nasal congestion, mostly affecting females at the age of around 30 years on average. Subsequently, nasal inflammation progresses to chronic eosinophilic rhinosinusitis, asthma, nasal polyposis, and intolerance to aspirin and to other NSAIDs. While it has been long established that NSAIDs cause inhibition of cyclooxygenase-1 (COX-1), leading to excessive metabolism of arachidonic acid (AA) to cysteinyl-leukotrienes (cys-LTs), there is now evidence that both cytokines and staphylococcus superantigens amplify the inflammatory process exacerbating the disease. This paper gives a brief overview of the development of chronic rhinosinusitis (CRS) in sensitive patients, and we share our experience in the diagnosis and management of CRS in AERD.

## 1. Introduction

The acute reaction to aspirin or other nonsteroidal anti-inflammatory drugs (NSAIDs) in aspirin-exacerbated respiratory disease (AERD) patients is a life-threatening condition characterized by upper airway symptoms (nasal obstruction and rhinorrhea) and lower airway symptoms (shortness of breath and respiratory distress). AERD patients, however, suffer from chronic manifestations of the disease including chronic rhinosinusitis, nasal polyps, and asthma—usually resistant to any treatment. The AIANE study showed that up to 80% of AERD patients required intermediate to high doses of inhaled steroids, and up to 50% of them had to take oral steroids in order to obtain asthma control. Similarly, the TENOR study showed that asthma was severe in 66% of AERD subjects, 34% received high systemic steroidal doses, 67% needed antileukotrienes, and 20% of them required orotracheal intubation during acute reactions. It must be noted that inflammation is usually present in both upper and lower airways and some AERD patients can present with a disease limited to the upper respiratory tract and no lower airways symptoms at all [1, 2]. Here, we review some of the most relevant aspects of rhinosinusitis in AERD.

## 2. Rhinosinusitis in AERD

Chronic rhinosinusitis is a major condition in AERD, which in its initial phases is manifested only as nasal congestion, with asthma starting about two years after the initial nasal symptoms. Chronic rhinosinusitis (CRS) is defined as an inflammatory condition involving the mucosa underlying the nasal cavity and the paranasal sinuses that can also affect the underlying bone. It usually lasts more than twelve consecutive weeks. In the case of AERD, CRS becomes a life-time condition, which is usually difficult to control. The clinical symptoms and signs to evaluate CRS are divided into major and minor criteria. The major criteria are nasal obstruction, facial pressure, nasal discharge, and/or postnasal drip. The minor criteria are the presence of purulence, anosmia and/or hyposmia, chronic cough, headache, dental pain, ear pressure, fatigue, and halitosis. The clinical evaluation is based on anterior rhinoscopy and nasal endoscopy that usually reveals mucosal oedema and hyperemia, with or without polyps, and frequently purulent secretions [3]. CRS can be divided into two mutually exclusive histological subtypes based on the presence of polyps or glandular hypertrophy. CRS with nasal polyps (CRSwNP) affects the full thickness of the nasal mucosa, which is replaced with an oedematous, generally eosinophilic, epithelium-coated “bag” of interstitial matrix “ground substance.” In contrast, CRS without nasal polyps (CRSsNP) or “hyperplastic rhinosinusitis” is characterized by glandular hypertrophy as demonstrated by Malekzadeh and colleagues [4, 5].

Eosinophils play an important role in the pathogenesis of CRS. Indeed, a greater number of these cells have been reported in both the upper and lower airways of patients of aspirin-sensitive patients as compared with aspirin-tolerant patients [6–8]. On activation, eosinophils release a vast array of mediators including leukotrienes, basic proteins (major basic protein and eosinophil cationic protein), cytokines, and oxygen-free radicals that cause local tissue damage. Saitoh et al. [9] found a correlation between the number of infiltrated eosinophils and both epithelial damage and BM thickening. CRSwNP is the most frequent form observed in aspirin-sensitive patients. We have found increased levels of eosinophil cationic protein in nasal secretions of aspirin-sensitive patients [10].

### 2.1. Nasal Polyps

**Figure 1:**
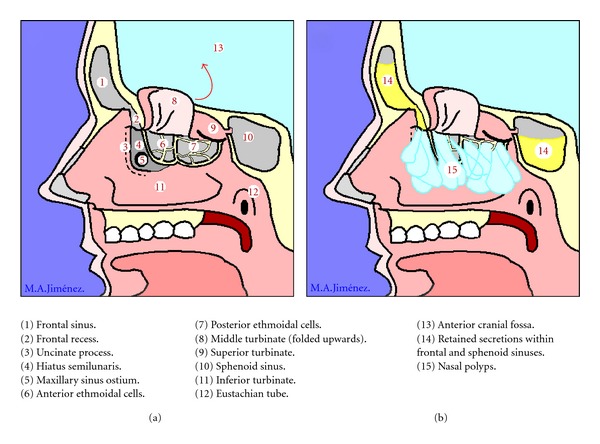
Schematic representation of (a) normal nasosinusal anatomy and (b) nasosinusal polyposis.

A majority of patients with aspirin intolerance will develop nasal polyps during the course of the disease. Nasal polyps are inflammatory pseudotumoral masses that most frequently start to grow from the ostiomeatal complex and the cells of the anterior ethmoidal sinus. They can affect the totality of the remaining sinusal cavities including the posterior ethmoidal cells, the maxillary, and the frontal or the sphenoidal sinuses, and they also can extend to the olfactory cleft, the sphenoethmoidal recess, and the nasal cavities (Figure 1). Nasal polyposis in AERD patients is present in up to 80 to 90% of patients and tends to be more aggressive and difficult to treat medically, also presenting with higher recurrence rates after surgery. In the AIANE study that included 500 ASA-intolerant patients from 14 different centres, nasosinusal polyposis was diagnosed on nasal endoscopy in 60% of the patients, but the prevalence rose to 90% when CT-scans of the nose and sinuses were performed [11].

Nasal polyps are formed by lax connective tissue stroma with oedema, inflammatory cells with a predominant eosinophil infiltration, mucosal glands, and newly formed vascular structures. The mucosal covering of the nasal polyps is a columnar glandular pseudostratified epithelium that also plays a major role in cytokine and inflammatory mediator release. There is no doubt that eosinophils are the main inflammatory cells found in the tissue specimens of nasal polyps, but neutrophils can also be present in large amounts and can even be predominant, notably in Asiatic patients. Other inflammatory cells can also be found in nasal polyps, such as lymphocytes, monocytes, plasma cells, and fibroblasts. Chronic sinusitis with nasal polyposis is also characterized by an intense oedematous stroma with albumin deposition, formation of pseudocysts, and subepithelial and perivascular inflammatory cell infiltration. Remodelling is a dynamic process regulated by diverse mediators among which TGF*β* is the most important. In addition to being a key factor in the generation or the deficit of T-reg cells, TGF*β* is also a critical factor implicated in the remodelling process in the airways through the attraction and proliferation of fibroblasts and upregulation of extracellular matrix synthesis. 

## 3. Epidemiology

The full prevalence of aspirin-induced rhinosinusitis can be difficult to assess, as it primarily relies on the clinical history. We believe that this condition is underdiagnosed worldwide and is only confirmed after an ASA challenge testing under ideal conditions. False negative results can occur when the patient is receiving systemic steroid or antileukotriene treatment. Systematic reviews report an incidence of 21.1% of ASA intolerance in asthmatic patients after ASA challenge testing. The incidence rises to 24% in patients with severe asthma and up to 40% when the association of asthma and chronic rhinosinusitis with polyposis is present [12–15].

**Figure 2:**
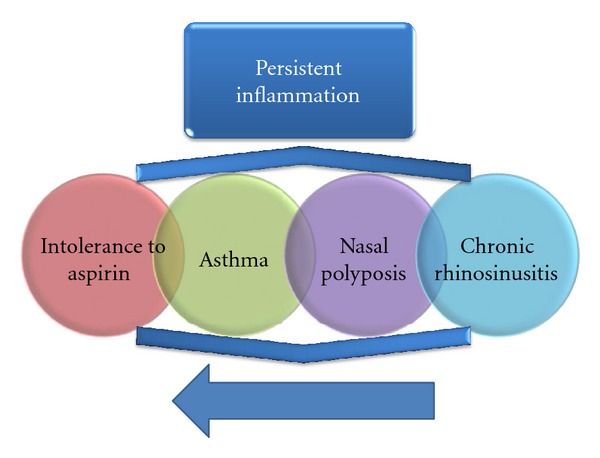
Natural history of aspirin-exacerbated respiratory disease.

Usually ASA intolerance appears in 30–40-year-old patients with a previous history of chronic rhinosinusitis and/or asthma. Some patients refer an acute viral-like nasal episode that never resolved fully afterwards. Nasosinusal symptoms usually progress to chronic sinusitis and nasal polyposis appears years later. Intrinsic asthma tends to appear after the rhinitis, but before the onset of nasal polyposis (Figure 2). 

Skin testing for allergy can be positive in up to 30–60% of AERD patients, but no association between ASA intolerance and IgE-mediated mechanisms has been identified. Nevertheless, a high prevalence of food intolerance or antibiotic allergies has been found [16–18].

## 4. Physiopathology

### 4.1. Arachidonic Acid

The physiopathologic mechanisms of AERD are still not fully understood. However, it is now well established that an alteration of arachidonic acid metabolism takes place, and it is characterized by an imbalance between cyclooxygenase (COX) and lipoxygenase pathways that results in an overactive lipoxygenase pathway. Thus, COX-1 inhibition after ASA or NSAIDs intake finally results in an overproduction of leukotrienes that leads to airway inflammation. Increased levels of leukotriene C_4_ synthase with elevated levels of LTC_4_, LTD_4_, and LTE_4_ with an overexpression of leukotriene producing enzymes such as 5-lipoxygenase (5-LO) and LTC_4_ synthase in nasal polyp tissue have been documented. Increased levels of urinary leukotriene metabolites have also been associated with hyperplasic CRSsNP and CRSwNP in both ASA-tolerant (ATA) and ASA-intolerant (AIA) patients. These urinary leukotriene metabolite levels tend to drop significantly after sinus surgery, probably as a consequence of the elimination of a great amount of leukotriene producing cells in the ethmoidal sinus. Indeed increased LTE_4_ levels and CysLT_1_ receptor overexpression have been identified in nasosinusal mucosa of aspirin-sensitive patients [19]. In an *in-vitro* study, Kowalski et al. [20] found that after incubation with 200 *μ*g of acetylsalicylic acid, 15-hydroxyeicosatetraenoic (15-HETE) was overproduced by nasal polyp epithelial cells and peripheral blood leucocytes of ASA-intolerant patients. Specificity and sensitivity of this test were both over 80%. 15-HETE has several proinflammatory effects such as inflammatory mediator release from the mast cells, mucosal glycoprotein release and bronchial smooth muscle contraction and thus could be a major trigger of airway inflammatory reactions. Supporting these observations, Mastalerz and colleagues show significant upregulation of some arachidonate lipoxygenation products in asthmatic subjects with aspirin hypersensitivity, as manifested by high baseline levels of 5-, 15-HETE in exhaled breath condensate [21].

The products of arachidonic acid also include lipoxins (LXs) and eoxins. The LXs modulate leukocyte trafficking and vascular tone, and in contrast to cys-LTs they have potent anti-inflammatory effects. A reduction in their levels may be a feature of AERD.

### 4.2. Cytokines

There has been a significant number of studies indicating that cytokines, in particular TH2 cytokines such as IL-4, regulate inflammatory processes in CRS. Increased IL-4 gene expression and IL-4 protein have been found in the upper airways in subjects with chronic sinusitis found as compared with controls. IL-4 promoter polymorphisms are also associated with nasal polyps in aspirin-sensitive patients. We have demonstrated that IL-4 is a major stimulus for eotaxin-2/CCL24 production. IL-13 and IFN-gamma also induce CCL24 production but they are less potent stimuli compared with IL-4 [22]. Pods et al. have reported increased expression of both eotaxin (CCL11) and eotaxin-2 in nasal polyps of patients suffering AERD, further supporting our findings in nasal polyposis [23]. The role of eotaxins in nasal polyps has been reviewed previously [24]. In a separate study, we have also shown that AERD patients with CRS release the eosinophil attractant CCL5 (RANTES) in nasal secretions, and lysine aspirin nasal challenge further increases the release of this chemokine. Consistent with this finding, Kupczyk et al. showed that these patients release increased levels of CCL5 and extended our observation by demonstrating the release of the eosinophil activating chemokine MCP-3. However, they did not show increased levels of CCL5 following lysine-aspirin challenge. We performed nasal challenge using a nitrocellulose filter, while they applied the nasal challenge by aerosol. These methodological differences may explain the differences observed in these two studies using the aspirin challenge. The release of CCL5 by nasal polyps has also been documented [25, 26].

IL-5 plays a prominent role in eosinophilic-driven processes, and it has been documented in CRSwNP in both aspirin-sensitive and aspirin-tolerant patients. A modest increase of IL-5 has been found in bronchial biopsies from AIA [27]. Interestingly, this cytokine is decisive regarding the impact of staphylococcus-aureus-(SE-) derived enterotoxins, which function as superantigens. S. aureus enterotoxin B (SEB) shifts the cytokine pattern further in nasal polyps toward T-helper-2 cytokines (increases interleukin-2, interleukin-4, and interleukin-5 greater than twofold), but it reduces the T-regulatory cytokines interleukin-10 and TGF-beta1. S. aureus-derived enterotoxins also influence local immunoglobulin synthesis and induce polyclonal immunoglobulin E production, which may contribute to severe inflammation via activation of mast cells [28]. Increased specific IgE to both S. aureus enterotoxin A (SEA) and SEB has been detected in nasal polyps from both subject groups, but median levels were markedly higher in AIA subjects than in ATA subjects [29].

Using a microarray cDNA technique, Sekigawa et al. [30] reported allelic associations of single nucleotide polymorphisms (SNPs) of INDO and IL1R2, in nasal polyps derived from aspirin-sensitive, but not aspirin-tolerant, patients. In contrast, Stankovic et al. identified periostin as a distinctive marker in nasal polyps derived from aspirin-sensitive patients [31].

Protein profiling is another approach, which has been used to investigate the pathogenesis of aspirin sensitivity in AERD patients. Zander et al. showed upregulation of *β*-adaptin and heat shock protein 70 (HSP70) in pooled nasal polyps samples from AIA using protein microarray [32]. *β*-adaptin has been reported to be one of many proteins that form complexes in clathrin-coated pits and vesicles during receptor-mediated endocytosis, while HSP70 seem to be involved in environmental stress as it occurs during the inflammation process indicating a greater degree of tissue damage. The increased expression of HSP70 may represent a cytoprotective adaptive response and result in altered cell regulation. This, in turn, may contribute to cellular proliferation and ultimately a more aggressive form of nasal polyposis refractory to treatment characteristically seen in aspirin-sensitive patients. 

### 4.3. Other Mechanisms

It has been proposed that autoimmunity may play a crucial role in AERD patients. However, it is possible that studies supporting this observation may reflect differences between ethnic groups as no association between AERD and autoimmunity markers has been proved [33].

Infection of the upper airways of AERD patients by anaerobic bacteria, Gram-negative organisms, staphylococcus aureus, and other bacteria is a frequent a complication. These bacteria are sources of antigens, which can form biofilms which in turn may amplify the inflammatory process. For instance, Bachert et al. demonstrated the presence of staphylococcus enterotoxins A and B in 50% of patients with polyposis in the homogenized polyp tissue. It is important to note that these same antigens also have the ability to stimulate the production of specific IgE antibodies [28].

Chronic viral respiratory infections have been suggested to play an important role in AERD via regulation of cytotoxic lymphocytes [34]. To date, however, there is not convincing evidence that this could be the case. Because the ethmoidal osteomeatal complex (OMC) in the nasal middle meatus is a site of deposition of toxic inhalants, viral particles, and airborne allergens, it is tempting to hypothesize that chronic infection of the ethmoidal sinus could play a role in the genesis of the inflammatory process in the upper airways of AERD patients. However, it does not explain the severity of asthma in these patients.

## 5. Diagnosis

The diagnosis of chronic sinusitis in AERD can occasionally be confusing in its early stages, since it usually precedes the onset of the AERD triad (asthma, ASA intolerance, and nasal polyposis) [35]. It can be easily confused with an allergic rhinitis although the skin tests for allergy will often be negative. However, either a nasal smear or blood count with high eosinophil numbers may give an initial diagnosis of nonallergic rhinitis with eosinophilia (NARES), an entity described in 1980 by Mullarkey et al. [36] and Jacobs et al. [37]. NARES has been suggested to be the precursor of nasal polyposis in both aspirin-tolerant and intolerant patients [38]. As the disease progresses, bronchial asthma and intolerance to aspirin will appear making the diagnosis of AERD easier (Figure 2). 

In contrast to the early diagnosis of rhinitis, the diagnosis of rhinosinusitis with polyps is much easier to perform. Symptoms such as hyposmia, nasal obstruction, anterior rhinorrhea, postnasal discharge, and occasionally cephalea are suggestive of nasal polyposis. Moreover, either anterior rhinoscopy or nasal endoscopy enables the examiner to see the protrusion of gray or pink translucent, multilobulated, nonfriable, and usually clustered tissue, which characterizes the polyps. A CT scan of the paranasal sinuses may be useful to determine the extension of the polyps in the sinusal cavities.

Aspirin challenge has been widely used to confirm the adverse reaction to NSAIDs. It is indicated in patients suffering asthma, rhinosinusitis, and nasal polyposis and in those with a history of near fatal reactions, but with negative history of ASA intolerance (15% of asthmatic patients with negative history may be intolerant to ASA). In patients with asthma and negative history of ASA intolerance, but with risk factors (rhinosinusitis, nasal, polyposis, a history of near fatal reactions) the risk increases and the test is totally required. Currently, there are four ASA challenges: oral, bronchial, nasal and intravenous. The oral challenge is considered the “gold standard”, and it is practiced mainly in the USA. In contrast, the aspirin bronchial inhalation challenge is used mainly in Europe. The aspirin nasal challenge is the safest since it induces bronchospasm rarely [39]. We use this last method as a diagnostic tool in our clinic on a routine basis.

Once AERD has been diagnosed, it is essential to assess whether it is associated with respiratory allergy. It has been proved that most patients with nasal polyps are sensitized against common allergens [40]. Therefore, all patients with AERD should undergo skin or serological tests as part of their initial evaluation and, if necessary, specific immunotherapy should be instituted.

## 6. Treatment

Undoubtedly, corticosteroids remain the cornerstone in the treatment of chronic hypereosinophilic sinusitis, whether accompanied or not by nasal polyposis [41–44]. Nasal topical steroids such as mometasone furoate, triamcinolone acetonide, or budesonide are used almost systematically as they have demonstrated a clear benefit in the control of mucosal oedema and of nasal polyposis. The prescription of topical nasal steroids is also very important for patients after endoscopic sinus surgery for nasal polyposis, as they can decrease the recurrence and growth of polyps [45]. Nasal lavages with saline solution or other commercial seawater sprays are also recommended to all patients. These should be performed at least twice a day in order to remove nasal secretions and crusts as much as possible before the application of the nasal steroid, thus facilitating its better absorption. 

Systemic steroids are also commonly used, notably in cases of moderate-to-severe nasal polyposis or poorly controlled asthma, since it has been demonstrated that after their administration there is, in addition to an improvement in FEV_1_, a decrease in polyp size and a reduction in both obstructive symptoms and rhinorrhea [46]. Some patients also show a partial or complete recovery of the sense of smell. It is generally recommended to administer short courses of prednisone calculated up to 1 mg/kg/day, with a maximum of 80 mg a day, which should always be taken in a daily single dose, early in the morning, in order to strengthen the circadian rhythm of endogenous cortisol. We do not recommend steroids in low doses for prolonged periods because of the long-term deleterious effects on the hypothalamic-pituitary-adrenal axis.

The prescription of antibiotics should be reserved only for cases of bacterial infection with the presence of purulent discharge and symptoms such as cephalea, rhinorrhea, posterior dripping, and fever. In such cases, the same antibiotics recommended for any acute bacterial nonpolypoid sinusitis are used empirically, specifically directed against haemophilus influenzae, streptococcus pneumonia, and moraxella catarrhalis. The recommended first choice antibiotic is amoxicillin in combination with clavulanic acid; some other options are second or third generation cephalosporins, macrolides, or fluoroquinolones. Some studies have demonstrated a beneficial effect with the administration of low doses of macrolides for prolonged periods in chronic sinusitis with or without nasal polyposis. In fact, it has been observed in some patients, especially of oriental races, that prolonged administration of low doses of these drugs may have an immunomodulatory effect leading to a reduction in the size of nasal polyps [47]. This immunomodulatory effect of macrolides is perhaps less pronounced in Caucasian patients, whose nasal polyps have a predominantly eosinophilic tissue infiltration, characteristic of an inflammation mediated by a TH2 response. 

The use of antileukotrienes has been considered an important adjunctive treatment of both bronchial asthma and chronic hypereosinophilic rhinosinusitis with or without polyps in the AERD [48]. However, both our experience and different reports in the literature show that their effect varies greatly from patient to patient. Their use can be justified by the overproduction of leukotrienes present in AERD. However, because of their cost and relative effectiveness, we do not consider them as a part of the primary treatment, and we prefer to reserve them for cases in which conventional treatment with nasal lavages, topical nasal steroids, and one to two short courses of systemic steroid per year are insufficient.

Aspirin desensitization may be beneficial in selected patients, but it must always be performed under supervised conditions. Several protocols of desensitization have been proposed allowing the completion of the procedure, usually within three to five days. The standard protocol for desensitization is an extension of the oral aspirin challenge protocol and all the safety precautions recommended for the challenge should be employed. Increasing aspirin doses (100–300 mg) are generally administered orally, although the intranasal administration is also a good alternative. Once desensitized, the patient must take a full dose of the prescribed amount of aspirin daily in order to maintain the desensitization effect. Up to 30% of patients will not tolerate the side effects of daily aspirin intake, and other COX-1 inhibitors such as ibuprofen can trigger bronchospasm in these individuals. It has been demonstrated that desensitization can improve asthma control and prevent the progressive growth of nasal polyps [49].

Although several biochemical events occur directly after achieving aspirin desensitization, such as downregulation of arachidonic acid metabolism, decreased inflammatory cell activation, downregulation in cysteinyl-leukotriene CysLT_1_ receptor expression, the real mechanism of aspirin desensitization remains unknown [50, 51]. Sweet et al. studied 107 AERD patients in a retrospective survey after 6 years of followup. Data from this study clearly demonstrated the clinical benefit of desensitization therapy for aspirin-sensitive patients, including a reduction in the number of hospitalization and emergency room visits, upper airway tract infections, sinus surgeries, and also, for many, an improvement in the sense of smell. However, twenty percent of the 65 patients of the therapy group reported upper digestive symptoms [52]. ASA desensitization is the only treatment that has shown clear impact on the natural course of AERD. Evidence suggests that desensitization reduces the growth and recurrence rate of nasal polyps in aspirin-sensitive patients in the long term. This indeed reduces the need for sinus surgery and allows a decrease in intranasal corticosteroid use. However, the impact of ASA desensitization on asthma has not been as consistent or reliable as the effects observed in the upper airway. 

Surgical treatment must also be considered when nasal polyposis fails to respond to medical treatment. Nowadays endoscopic sinus surgical techniques are preferred because of their proven safety and reduced morbidity. Several studies confirm that endoscopic sinus surgery in AERD patients not only improves nasal symptoms but also enables a better control of asthma [53]. Therefore, surgical treatment should be considered in all cases of nasosinusal polyposis when medical treatment fails to improve nasosinusal symptoms, when more than two short courses of systemic steroids per year are needed, or when asthma is not controlled. Another special indication for surgery is anosmia or hyposmia, and even more so in patients with “professional noses” such as chefs, winemakers, sommeliers, and perfume creators. However, the impact of surgery on olfaction is never guaranteed and surgery by itself could increase olfactory losses. 

Currently, there are three types of surgery indicated for nasal polyposis. In simple polypectomy, only the visible polyps in the nasal cavities are removed, without penetrating into the ethmoidal cells. Functional ethmoidectomy includes the opening of all the affected ethmoidal cells, with the resection of all polyps, but with minimal removal of the ethmoidal mucosa. Finally, the nasalisation technique consists of a complete bilateral ethmoidectomy with eradication of all the ethmoidal mucosa, except the one covering the frontal recess boundaries. Wide antrostomy and sphenoidotomy are also performed, and when needed, the middle turbinate can also be included in the resection [54]. The choice of surgical technique will certainly depend on the preference and personal training of each surgeon; however, increasing reports in the literature favor a more radical removal of the ethmoidal mucosa. In fact, it has been demonstrated that limited ethmoidectomy without mucosal removal offers long-term results comparable to those of a simple polypectomy; therefore, we do not consider that the risk of a partial ethmoidectomy is justified if a more limited and more secure procedure can achieve similar results [55]. Nasalisation appears to offer better long-term results with a lower rate of recurrence of polyposis, less nasal obstruction and an improvement of olfaction that is more stable over time. It is also noteworthy that a radical surgery has a more positive and prolonged impact on the stabilization of bronchial asthma both in ASA-tolerant and intolerant patients [56]. Jankowski et al. compared nasalisation with conventional ethmoidectomy, proving that a more radical surgery on the ethmoid mucosa does offer better functional results, both on olfaction and general nasal function [56–58]. Some authors actually advocate the systematic resection of the middle turbinate, since this provides a more complete removal of the ethmoidal mucosa, resulting in a lower incidence of polyposis recurrence in the long term. However, based on our experience, we believe that the middle turbinate is an important anatomical landmark that is helpful in cases of revision surgery, and also, with its preservation, frontal recess stenosis is less likely to occur. Thus, we prefer to resect the middle turbinate only when it is affected severely by polypoid disease or when it has lost its structural support after the ethmoidectomy. In this latter situation, the resultant middle turbinate lateralization could occlude the surgical cavity, thereby impairing sinus lavages or topical steroid penetration and causing polyp relapse and chronic sinusitis.

The surgical goals of nasalisation are the eradication of the ethmoidal mucosa and the creation of a cavity as wide as possible, in order to facilitate the subsequent cleaning and the proper dissemination of topical steroid nasal spray [59]. It is also important to promote good mucosal healing through immediate postoperative application of a parenteral depot systemic steroid and the early reinstitution of topical nasal steroid use. Clinically, the goals of surgery are first, improvement of nasal obstructive symptoms with, reduced rhinorrhea and postnasal discharge; second, a stable and sustained improvement of smell; finally, a better control of bronchial asthma. Despite all the medical and surgical treatments described above, there are cases in which stabilizing a patient with AERD is extremely difficult. Some studies have reported cases in which methotrexate proved to be useful [60].

## 7. Conclusion

Despite all the major advances in research, understanding fully the mechanism of AERD still remains a challenge to the modern medical world. It is now well established that aspirin causes inhibition of cyclooxygenase-1 (COX-1) associated with excessive metabolism of arachidonic acid (AA) to cysteinyl-leukotrienes (cys-LTs), while there is a rapid decrease in the synthesis of COX-1 products including prostaglandin E_2_, which is known to have bronchodilator and anti-inflammatory properties. Moreover, cytokines and staphylococcus superantigens further amplify the inflammatory process in the airways of AERD patients. Chronic treatment with moderate-to-high doses of inhaled steroids is indicated. However, it is important to keep in mind that a multidisciplinary approach is the cornerstone for a successful treatment of these patients.
